# From Nonalcoholic Fatty Liver Disease to Metabolic Dysfunction-Associated Steatotic Liver Disease: Out with the Old, in with the New

**DOI:** 10.3390/jcm13030880

**Published:** 2024-02-02

**Authors:** Iyiad Alabdul Razzak, Mazen Noureddin, Hirsh D. Trivedi

**Affiliations:** 1 Department of Medicine, St. Elizabeth’s Medical Center, Tufts School of Medicine, Boston, MA 02111, USA; 2 Houston Research Institute and Houston Methodist Hospital, Houston, TX 77030, USA; 3 Comprehensive Transplant Center, Cedars-Sinai Medical Center, Los Angeles, CA 90048, USA; 4 Karsh Division of Gastroenterology, Cedars-Sinai Medical Center, Los Angeles, CA 90048, USA

## 1. Introduction

Nonalcoholic fatty liver disease (NAFLD) is the most common cause of chronic liver disease (CLD) affecting a quarter of the global population [1,2]. NAFLD-related cirrhosis ranks as the second-most common reason for liver transplants in the United States, underscoring its role as a major health and economic burden [1,2,3]. Consequently, NAFLD has been a prominent focus in research and patient awareness campaigns [4]. Despite that, the nomenclature describing this condition and its related spectrum of liver diseases has several limitations, viewed from the perspectives of both physicians and patients [5,6]. These limitations ultimately led three pan-national liver associations to initiate a modified Delphi process aiming to find a new nomenclature and definition that, to the greatest extent possible, would address all the shortcomings of the previous nomenclatures. This effort led to the birth of a new consensus-based set of terminologies and definitions under the umbrella of steatotic liver disease (SLD) [6]. Adoption of the new nomenclature, however, has been variable. Herein, we aim to summarize and simplify the new changes.

## 2. Evolution of Nomenclature

After the recognition of the link between obesity and liver steatosis with hepatic inflammation, the term ‘nonalcoholic steatohepatitis’ (NASH) surfaced in 1980 [7]. Then, ‘NAFLD’ was used to describe the histological spectrum from steatosis to steatohepatitis [6]. NAFLD was defined as hepatic steatosis affecting at least 5% of hepatocytes in those who consume little or no alcohol without any secondary causes of liver disease [8]. Since then, NAFLD has become more prevalent and recognized, leading to increased research and understanding of this disease [9]. Subsequently, efforts have been made to develop targeted therapeutics [10,11]. During this process, multiple limitations of the term NAFLD have become evident [5,6]. These include the inaccuracy of describing disease etiology when using the exclusionary term ‘nonalcoholic’; the potential stigma of terms like ‘alcoholic’ and ‘fatty’; the omission of metabolic risk factors in the disease definition; and the failure to account for the coexistence of other etiologies of CLD, particularly alcohol-related liver disease (ALD) [5,6,12]. As a result, over recent years, efforts have been made to establish an alternative nomenclature that has global consensus. In 2020, Eslam et al. introduced the term ‘metabolic dysfunction-associated fatty liver disease’ (MAFLD), which was defined as fatty liver with metabolic risk factors regardless of alcohol intake [5]. This was viewed as a step forward by using ‘positive’ rather than exclusionary criteria by correctly replacing ‘nonalcoholic’ with ‘metabolic-dysfunction’. Nevertheless, concerns emerged about the sole reliance on metabolic risk factors, which permits a more liberal alcohol use, the potentially stigmatizing word ‘fatty’, and the tendency for the spectral nature of this disease to be overlooked [6,13]. As such, establishing a new, globally unified nomenclature and diagnostic criteria that address the previously mentioned limitations has become paramount.

## 3. Delphi Consensus Statement on New Nomenclature

In late 2021, a modified Delphi process was initiated by three large pan-national liver associations: the American Association for the Study of Liver Diseases (AASLD), the European Association for the Study of the Liver (EASL), and the Asociación Latinoamericana para el Estudio del Hígado (ALEH) [6]. More than 200 experts in fields relevant to NAFLD from over 50 countries were engaged in this process. The aim was to determine whether participants favored a change in nomenclature and/or definition and to develop a consensus on new nomenclature and diagnostic criteria for this condition. A Steering Committee, composed of two co-chairs representing AASLD and EASL plus 34 members nominated by their respective pan-national societies and patient advocacy organizations, developed a set of consensus statements. A total of 236 panelists participated in four rounds of online surveys and two meetings (April 2022 to January 2023) to refine the statements and determine consensus, defined a priori as the supermajority (67%) vote. Final consensus statements were based on four key factors: the desire for a name change and the role of stigma; the structure of a new name; the new disease definition; and the perceived impact on disease awareness, biomarker development, and clinical trials. Seventy-four percent of respondents agreed on the need for a name change. The terms ‘nonalcoholic’ and ‘fatty’ were felt to be stigmatizing by 61% and 66% of respondents, respectively. Steatotic liver disease (SLD) was chosen as an overarching term encompassing various causes of steatosis. The term metabolic dysfunction-associated steatotic liver disease (MASLD) was chosen to replace NAFLD [6], while the term non-alcoholic steatohepatitis was retained under the new name ‘metabolic-dysfunction associated steatohepatitis (MASH)’ given its importance as a separate disease stage [6].

## 4. The New Nomenclature: All You Need to Know

**Figure 1:**
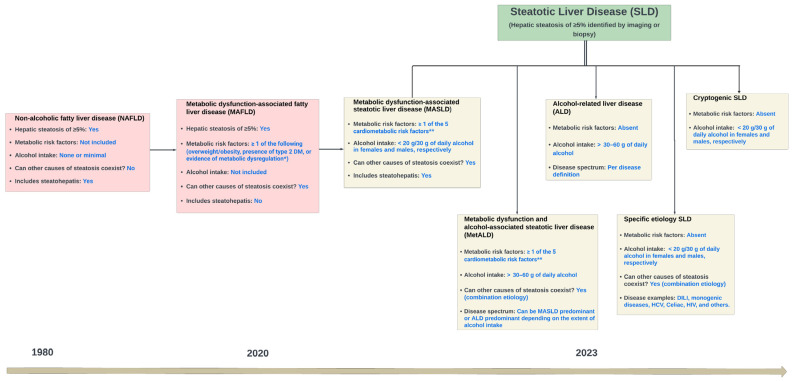
The evolution of metabolic dysfunction-associated steatotic liver disease (MASLD) nomenclature and disease definition. This also depicts the newly established steatotic liver disease (SLD) and its subcategories. * Metabolic dysregulation is defined as the presence of at least two of the following criteria: waist circumference ≥ 102/88 cm in Caucasian men/women (or ≥90/80 cm in Asian men/women), respectively; blood pressure ≥ 130/85 mmHg or specific drug treatment; plasma triglycerides ≥ 150 mg/dL or dyslipidemia drug treatment; plasma HDL-cholesterol < 40 mg/dL for men and <50 mg/dL for women; prediabetes; homeostasis model assessment of insulin resistance score ≥ 2.5; plasma high-sensitivity C-reactive protein level > 2 mg/L. ** The five cardiometabolic risk factors are (1) body mass index (BMI) ≥ 25 kg/m^2^ (≥23 kg/m^2^ for Asians), waist circumference > 94 cm for males and >80 cm for females, or ethnicity-adjusted; (2) fasting serum glucose ≥ 100 mg/dL, 2 h post-load glucose levels ≥ 140 mg/dL, hemoglobin A1c ≥ 5.7%, type 2 diabetes, or treatment for type 2 diabetes; (3) blood pressure ≥ 130/85 mmHg or specific antihypertensive drug treatment; (4) plasma triglycerides ≥ 150 mg/dL or lipid-lowering treatment; (5) plasma HDL cholesterol ≤ 40 mg/dL for males and ≤50 mg/dL for females or lipid-lowering treatment. Abbreviations: HIV, human immunodeficiency virus; HCV, hepatitis C virus; DILI, drug-induced liver injury.

The new nomenclature, disease definition, and classification addressed the limitations of the former nomenclatures. Firstly, the new MASLD term replaced exclusionary and potentially stigmatizing terms (‘nonalcoholic’ and ‘fatty’) with more etiologically accurate and non-stigmatizing terms (‘metabolic-dysfunction’ and ‘steatotic’, respectively) [6,14]. Secondly, using SLD as an overarching term provides a clear framework for categorizing different etiologies of hepatic steatosis. Thirdly, the new definition employs two simple criteria to diagnose MASLD in patients with hepatic steatosis: (1) the presence of one or more cardiometabolic risk factors, and (2) the absence of significant alcohol intake (>20 g/30 g of daily alcohol in females and males, respectively) or other causes of steatosis [6]. Additionally, a new category named metabolic dysfunction and alcohol-associated steatotic liver disease (MetALD) was introduced to separate an important group of patients—those who have one or more cardiometabolic risk factors and consume more than 30–60 g of alcohol daily [6]. The introduction of the MetALD spectrum was prompted by the role of alcohol in altering the disease’s natural history and response to treatment [6,15]. Finally, ‘cryptogenic SLD’ was introduced as an interim label for cases of steatosis without CMRFs or other identifiable causes of steatosis, pending further advancements in our understanding of disease pathophysiology [6]. Figure 1 depicts the evolution of the MASLD nomenclature and the difference between the old and new disease definitions. 

## 5. Advantages, Future Implications, and Challenges of the New Nomenclature

The new term MASLD, under the umbrella of SLD, represents a positive shift in the context of the most common liver disease worldwide, while also presenting potential future challenges. An important consideration involved in developing this nomenclature was to ensure the preservation and to build upon existing NAFLD data without hindering ongoing biomarker and drug development research [6,14]. A recent large cohort analysis showed a 98% overlap between patients with conventional NAFLD and those with the newly suggested MASLD [6,16]. This practically allows for the generalization of NAFLD data to MASLD. In addition, preserving steatohepatitis by replacing NASH with MASH reduces confusion in clinical practice and drug trials, as the latter have ‘resolution of steatohepatitis’ as a common endpoint for approval [14]. The affirmative and non-stigmatizing nature of the new terms are expected to help increase disease awareness and involve a broader range of patients [14,17]. Finally, the new definition is anticipated to simplify the diagnostic process, making it more straightforward and intuitive [14]. 

The new nomenclature comes with few unexplored areas. Firstly, categorizing MetALD as a continuum, coupled with our limited understanding of the interplay between metabolic dysfunction and alcohol intake on SLD development, may pose challenges in developing disease-specific biomarkers or drugs for this group. Secondly, the new nomenclature lacks clarity regarding the placement of lean NAFLD patients with no other cardiometabolic risk factors. Nonetheless, it should be noted that these scenarios are rare, and the new nomenclature does provide room for adding new subtypes under ‘cryptogenic SLD’ depending on future research [6]. Finally, while the new term has gained acceptance among experts worldwide, the transition from the long-established NAFLD to MASLD appears to vary with slowness particularly in the broader community. Rotonya M. Carr proposes a ten-step strategy to help clinicians promote widespread adoption of the new nomenclature [18]. 

In conclusion, NAFLD has been replaced with MASLD, simplifying the lives of both clinicians and patients. It is worth taking a moment to understand the evolution of the new terminology in order to promote its widespread adoption.
